# Does the Primary Care Experience Influence the Cancer Diagnostic Process?

**DOI:** 10.1155/2015/176812

**Published:** 2015-10-04

**Authors:** Sylvie Provost, Raynald Pineault, Pierre Tousignant, Danièle Roberge, Dominique Tremblay, Mylaine Breton, Lynda Benhadj, Mamadou Diop, Michel Fournier, Astrid Brousselle

**Affiliations:** ^1^Direction de Santé Publique, Centre Intégré Universitaire de Santé et Services Sociaux du Centre-Sud-de-l'Île-de-Montréal, 1301 rue Sherbrooke Est, Montréal, QC, Canada H2L 1M3; ^2^Centre de Recherche du Centre Hospitalier de l'Université de Montréal, Hôtel-Dieu, Pavillon Masson, 3480 rue Saint-Urbain, Montréal, QC, Canada H4W 1Y1; ^3^Institut de Recherche en Santé Publique de l'Université de Montréal, 7101 avenue du Parc, Montréal, QC, Canada H3N 1X9; ^4^Institut National de Santé Publique du Québec, 945 avenue Wolfe, Québec, QC, Canada G1V 5B3; ^5^Department of Epidemiology, Biostatistics and Occupational Health, McGill University, 1020 avenue des Pins Ouest, Montréal, QC, Canada H3A 1A2; ^6^Centre de Recherche de l'Hôpital Charles-LeMoyne, Université de Sherbrooke, Campus de Longueuil, 150 place Charles-LeMoyne, Longueuil, QC, Canada J4K 0A8; ^7^Département des Sciences de la Santé Communautaire, Faculté de Médecine et des Sciences de la Santé, Université de Sherbrooke, Pavillon Gérald-Lasalle, 3001 12^e^ avenue, Sherbrooke, QC, Canada J1H 5H3; ^8^École des Sciences Infirmières, Faculté de Médecine et des Sciences de la Santé, Université de Sherbrooke, Pavillon Gérald-Lasalle, 3001 12^e^ avenue, Sherbrooke, QC, Canada J1H 5H3

## Abstract

*Objective*. To analyze the impact of patients' experience of care at their usual source of primary care on their choice of point of entry into cancer investigation process, time to diagnosis, and presence of metastatic cancer at time of diagnosis. *Method*. A questionnaire was administered to 438 patients with cancer (breast, lung, and colorectal) between 2011 and 2013 in four oncology clinics of Quebec (Canada). Multiple regression analyses (logistic and Cox models) were conducted. *Results*. Among patients with symptoms leading to investigation of cancer (*n* = 307), 47% used their usual source of primary care as the point of entry for investigation. Greater comprehensiveness of care was associated with the decision to use this source as point of entry (OR = 1.25; CI 90% = 1.06–1.46), as well as with shorter times between first symptoms and investigation (HR = 1.11; *p* = 0.05), while greater accessibility was associated with shorter times between investigation and diagnosis (HR = 1.13; *p* < 0.01).  *Conclusion*. Experience of care at the usual source of primary care has a slight influence on the choice of point of entry for cancer investigation and on time to diagnosis. This influence appears to be more related to patients' perceptions of the accessibility and comprehensiveness of their usual source of primary care.

## 1. Introduction

Longer time to diagnosis of cancer may be detrimental in many ways, resulting in a more advanced stage at diagnosis, increased morbidity, psychological distress, and reduced survival [1]. Conversely, shortening that time could result in diagnosis at earlier stages and improved patient prognosis [2–4], especially in cases of colorectal and breast cancer [1]. Most cancer diagnoses are established among symptomatic patients consulting in primary care clinics [5–7], and primary care physicians can play a key role in shortening time to diagnosis [8].

Despite a lack of consensus on the definition and measurement of the prediagnostic period [2, 9–16], the elapsed time before cancer diagnosis can be subdivided into several segments [2, 17–19], such as elapsed time between first symptoms and start of investigation and between start of investigation and diagnosis. These times vary depending on, among other things, type of cancer and sociodemographic characteristics of patients [12, 17, 19–23], as well as on symptom specificity and patients' anxiety level [12, 23]. Patient-related factors are more likely to influence part of the elapsed time from first symptoms to start of investigation, whereas certain factors related to health system organization are likely to influence not only part of the time from first symptoms to start of investigation but also the time from start of investigation to diagnosis, and potentially have an impact on cancer stage at time of diagnosis [18, 19]. This is the case with respect to patients' experience of care at their usual source of primary healthcare (PHC) and to their choice of point of entry into the investigation process leading to cancer diagnosis.

Several studies have demonstrated the impact of PHC services organization on patients' experience of care and other aspects of health services utilization [24–28]. Commonwealth Fund data compiled from several countries show considerable variation in patients' reported experience of care in terms of accessibility, continuity, comprehensiveness, care coordination, and chronic illness management [29]. Canada is generally within the average range, and Quebec is generally below the Canadian average. However, to our knowledge, few studies have specifically explored the impact of primary care experience on the cancer diagnostic process. The study by Rogers et al. [30] appeared to indicate that confidence in the primary care physician played a greater role than accessibility in the cancer detection process. The findings of Hansen et al. [31] suggested that perceived greater accessibility of primary care physicians and patients' higher levels of confidence in their physicians were associated with shorter times between first symptoms and primary care consultation. Also, to our knowledge, there has been no study published on the impact of the experience of care at the usual source of PHC on patients' choice of point of entry into the cancer investigation process. This question is of great importance in a healthcare system such as Quebec's where, although consulting first a family physician in a PHC setting is advocated for most health problems, patients may choose their point of entry into the care process and have access directly to specialists. Moreover, since in Quebec the cancer investigation process may be initiated by the primary care physician or by a specialist after referral, experience of care at the usual source of PHC may have an impact on the duration of the diagnostic process.

The objective of the present study was to analyze the impact of the care experience at the usual source of PHC on (1) choice of point of entry into the investigation process leading to cancer diagnosis, (2) time to diagnosis, and (3) presence of metastatic cancer at time of diagnosis.

## 2. Methods

### 2.1. Design

**Figure 1:**
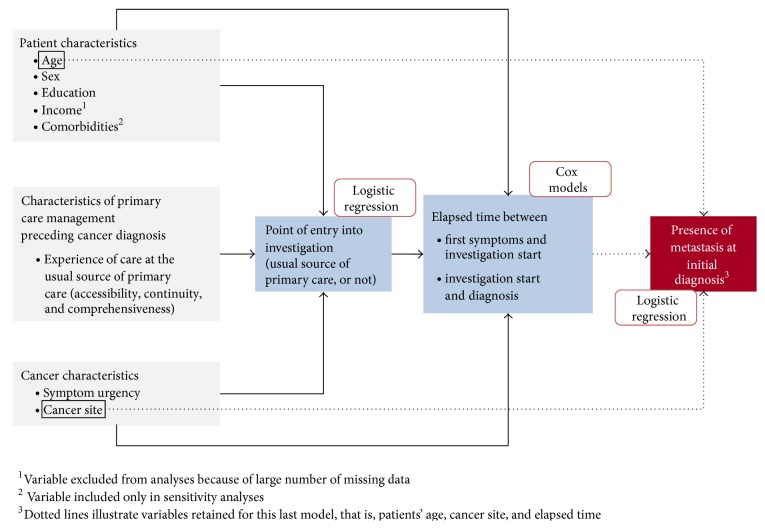
Model for analysis of factors associated with choice of point of entry into the cancer diagnostic process, time to diagnosis, and presence of metastasis at time of diagnosis.

This cross-sectional exploratory study consisted of a survey conducted between 2011 and 2013 of adult patients with breast, lung, or colorectal cancer. Patients invited to participate in the study (convenience sample) had been diagnosed with a first cancer and enrolled for less than three months in a cancer clinic at one of four participating hospitals in two of Quebec's most populous regions (Montreal and Montérégie). The analyses presented in this paper relate to patients who had symptoms leading to investigation of their cancer. The analysis model (Figure 1) was inspired by the Behavioral Model of Health Services Use [32–34]. It includes predisposing factors (patient characteristics) and disease characteristics (cancer site and symptom urgency). The choice of point of entry into investigation and the elapsed time in the investigation process characterize the cancer diagnostic process and reflect services use.

### 2.2. Source of Data

In order to analyze the phenomena from the patient perspective, data were collected from patients by means of a self-administered questionnaire that documented their affiliation with a usual source of PHC, their experience of care at that source, their use of health services in the period preceding their cancer diagnosis, sociodemographic characteristics, and presence of comorbidities. The questionnaire, which took about 15 minutes to complete, consisted of closed-ended questions and some semiopen questions (dates). It was validated with physicians and pretested on 10 oncology clinic patients. For each patient participating in the study, clinical data on cancer site and presence of metastasis at time of diagnosis were obtained from the cancer registry of each participating hospital.

### 2.3. Key Variables

The* point of entry* into the cancer investigation process was the place where a physician prescribed the first tests that led to the patient's cancer diagnosis. This could be, for instance, the patient's usual source of PHC, another PHC clinic, a hospital emergency room, or a specialist.

The* time *(or* elapsed time*) to cancer diagnosis was calculated based on dates provided by the patient in the questionnaire: date when the patient began to have unusual symptoms or signs that could now be attributed to the cancer; date when the patient made an appointment with a physician for these unusual signs or symptoms; date when the physician prescribed tests to diagnose the cancer; and date when the patient received the cancer diagnosis. Patients who were unable to provide specific dates were asked to indicate the month and year of the event and whether the event occurred at the beginning, middle, or end of the month; the mid-point of each of these three periods was then used as the event date. Residual missing data for date of diagnosis were estimated from the dates of diagnosis entered in the oncology clinics' cancer registries, adjusted to take into account the mean difference between dates of diagnosis entered in the registries and dates given by responding patients, by cancer site.  Appendix A presents the percentages of data that were attributed.

Data on* presence of metastasis* at time of initial diagnosis were extracted for each participating patient from the oncology clinic cancer registries.* Cancer site* (breast, lung, and colorectal) was identified by means of a question in the patients' questionnaire and confirmed by data from the oncology clinic cancer registries.

The section of the questionnaire dealing with* experience of care* was constructed based on various validated instruments, in particular the Primary Care Assessment Survey (PCAS) and the Primary Care Assessment Tool (PCAT) [35–37]. Adaptation of these instruments to the context of healthcare system in Quebec required some adjustments and has been validated in two studies conducted in Quebec in recent years [38, 39]. Three indices of experience of care at the usual source of PHC were constructed based on 13 indicators emerging from the questionnaires: accessibility of services, continuity of care, and comprehensiveness. Accessibility refers to the ease with which people are able to use services, particularly with regard to temporal and organizational barriers to use; continuity describes the phenomenon of flow and interruption in a temporal sequence over the course of which several services must be provided; and comprehensiveness assesses the response by the usual source of PHC to all the patient's health services needs [40]. The list of indicators making up the care experience indices can be found in Appendix B. The composite indices, validated in previous studies [38, 39], were constructed following a formative approach [41, 42]. The items were not submitted to factor analysis, which is inappropriate in the formative approach because the items are not necessarily correlated [41, 42]. The indices constructed were expressed as scores on a scale of 0 to 10 points. Details on the construction of the indices are available upon request from the authors. It should be noted that, in this study, the usual source of PHC is defined as the PHC clinic where, in the two years preceding the cancer diagnosis, the person usually or most often went to see a physician for general care.

*Symptom urgency* was determined, by expert (physician) consensus, from patients' responses to an open-ended question asking them to describe the unusual signs or symptoms that led them to consult a physician and that they or their physician could now attribute to the cancer. Symptoms were considered urgent if it would have been appropriate for the patient to consult elsewhere (e.g., another PHC clinic, emergency room) in case the appointment with the usual source of PHC was too far off in time.

### 2.4. Data Analysis

Multiple regression analyses were used to measure the association between experience of care at the usual source of PHC and the various outcomes under study. Based on the general analysis model (Figure 1), three regression models were constructed. A first logistic regression model was used to measure the association between experience of care and correspondence between usual source of PHC and point of entry into investigation, controlling for both patient and cancer characteristics. Cox models were used to measure the association between experience of care and time to cancer diagnosis, controlling for patient characteristics, cancer characteristics, and point of entry into the investigation process. For the Cox regression regarding first symptoms-to-investigation time, the origin of the time-scale of the analysis was the date of first symptoms and the exit point was the start of investigation. For the analysis of investigation-to-diagnosis time, the origin of the time-scale of the analysis was the start of the investigation and the exit point was the disclosure of the diagnosis. Lastly, a logistic regression model was used to measure the influence of elapsed time prior to cancer diagnosis on the presence of metastatic cancer at time of diagnosis, controlling for patient age and cancer site. The analyses were performed using STATA software, version 13.1.

### 2.5. Ethical Considerations

The project received ethical approval from the four participating hospitals and from the Ethics Committee of the Charles-LeMoyne Hospital Research Centre.

## 3. Results

Overall, 438 patients accepted to participate in this study (response rate of 85%). Of the 438 patients recruited, 307 (70.1%) had presented symptoms that led to investigation for cancer; in the others, cancer was detected either as the result of a screening test or inadvertently during investigation of another health problem. Of these 307 patients, 34.9% had breast cancer, 29.6% had lung cancer, and 35.5% had colorectal cancer (Table 1). Nearly 70% were women, and more than one-third were 65 years of age or older. More than half of the patients had completed college or university. A large proportion reported presenting at least one comorbidity: 48.4% had at least one cardiometabolic risk factor and 50.0% had at least one chronic illness. More than one-quarter of the patients presented metastases at the time of initial cancer diagnosis.

The large majority of patients (89.3%) had a usual source of PHC in the two years preceding their cancer diagnosis (Table 2). The experience of care at this source was generally positive; however, continuity and comprehensiveness scores were higher than accessibility scores.

In nearly half the cases (46.9%), the point of entry into the cancer investigation process was the usual source of PHC (Table 3). The hospital emergency room was the point of entry for one-quarter of patients with lung or colorectal cancer. The main reasons why patients with a usual source of PHC did not consult that source (*n* = 126) were to be able to undergo the required tests within a reasonable time frame (31.8%), to be able to consult a specialist within a reasonable time frame (30.2%), because it was difficult to obtain an appointment within a reasonable time frame (18.3%), or because their condition was too urgent (12.7%). The last reason was mentioned more often by patients with lung or colorectal cancer (16.2% and 20%, resp.) than by those with breast cancer (2.3%).

The median elapsed time between the appearance of the first symptoms triggering the cancer investigation and the medical appointment marking the start of the investigation was 47 days, and the median elapsed time from that medical appointment to the disclosure of the cancer diagnosis was 32 days (Table 4). The latter period was significantly longer (*p* = 0.09) for breast cancer than for lung or colorectal cancer.

Regardless of cancer site, lower symptom urgency was associated with using the usual source of PHC as the point of entry into the cancer investigation process (Table 5). With regard to experience of care, patients with more positive perception of the comprehensiveness of care at their usual source of PHC were more likely to have used that source as the point of entry into the cancer investigation process.

The results of the multivariate analyses presented in Table 6 indicate that when controlling for cancer site, age, sex, education level, urgency of first symptoms prompting cancer investigation, and point of entry into the investigation process, patients whose experience of comprehensiveness of care at their usual source of PHC was more positive had shorter times between first symptoms and start of investigation (hazard ratio 1.11; *p* = 0.05). Elapsed time between start of investigation and diagnosis was observed to have been shortest for patients with lung cancer (hazard ratio 1.46; *p* = 0.03). Patients with greater accessibility to their usual source of PHC, as well as those that are with no usual source of PHC or whose point of entry into the investigation process was not their usual source of PHC, also had shorter investigation-to-diagnosis times (hazard ratios 1.13, 4.00, and 1.40, resp.).

Multivariate analyses showed that patients with lung or colorectal cancer were more likely to have metastases at time of diagnosis (Table 7). In our analyses, no significant association was seen between symptoms-to-investigation time or investigation-to-diagnosis time and the presence of metastases at time of diagnosis.

## 4. Discussion

Our results indicate that the experience of care at the usual source of PHC exerts a modest but statistically significant influence on the choice of point of entry into the process leading to cancer diagnosis and on time to diagnosis. This impact of experience of care seems to be linked more to the dimensions of accessibility and comprehensiveness than to care continuity. In effect, greater comprehensiveness of care was associated with using the usual source of PHC as the point of entry and with shorter symptoms-to-investigation times, and greater accessibility was associated with shorter investigation-to-diagnosis times.

### 4.1. Point of Entry into the Cancer Investigation Process

This study's findings shed light on the choice of point of entry. Even when patients had a usual source of PHC, in about half the cases this was not their point of entry into the cancer investigation process. Lower symptom urgency was strongly associated with using the usual source of PHC as the point of entry into the cancer investigation process, suggesting that when patients present a more urgent or severe condition, they feel bypassing their usual source of PHC may accelerate the diagnostic process. Likewise, some of the reasons mentioned by patients who had not chosen their usual source of PHC as the point of entry reflected problems of accessibility related to this source (e.g., difficulty obtaining an appointment within a reasonable time frame). Other reasons evinced a desire to accelerate the investigation process, such as by going directly to the technical platform or specialist services (e.g., to undergo the necessary testing or see a specialist within a reasonable time frame). In some cases, patients reported that their health condition required immediate recourse to emergency services. Nevertheless, we observed that, after controlling for patient and cancer characteristics in the multivariate analyses, comprehensiveness of care was the only component of patients' primary care experience associated with their choice of using their usual source of PHC as the point of entry into the cancer investigation process. Patients' perception that their usual source of PHC was responsive to all their concerns (i.e., it looked after all their health issues, whether physical or psychological, and helped them obtain all the services they required) thus appeared to play an important role in patients' choice of point of entry.

### 4.2. Time to Cancer Diagnosis

The comprehensiveness of care received at the usual source of PHC was also associated with shorter symptoms-to-investigation times. As mentioned, this time actually comprises two periods. The period from the first recognition of symptoms to scheduling an appointment is largely influenced by the nature or urgency of the symptoms [12, 23] and patients' characteristics [17, 20], some of which were not measured in our study. On the other hand, the period from scheduling an appointment to the physician consultation that marked the start of investigation is more likely to be associated with patients' experience of care at their usual source of PHC. Because of the large number of missing data with regard to the date of scheduling the appointment, we were not able to analyze these periods separately. The association between comprehensiveness of care and a shorter symptoms-to-investigation time suggests that patients' perception of their usual source of PHC being responsive to all their health concerns influences them positively to contact that source more promptly in the case of worrisome symptoms, even if accessibility might not be optimal. This association might also reflect the fact that patients who are followed up for all their health problems at their usual source of PHC may obtain appointments sooner than others when new symptoms arise. The study by Rogers et al. [30], based on ecological analysis of both survey data from PHC clinics and administrative data on referrals of cancer patients to an urgent (2-week-wait) referral system, indicated that patients' confidence in their physician played a greater role than did accessibility in the cancer detection process. It is possible that the perception of care comprehensiveness, as measured in our study, reflects patients' level of confidence in their usual source of PHC.

With regard to investigation-to-diagnosis time, our results revealed shorter times among patients whose usual source of PHC was more accessible. Particularly in cases where the source of PHC plays a coordinating role in the investigation process, greater accessibility can, in fact, translate into a succession of shorter times over the different stages of the process. In the study by Hansen et al. [31], times were shorter among patients who were seen by doctors who knew them less well and who worked in PHC clinics offering the widest range of services, characteristics often associated with more accessible clinical settings. In our study, the fact that patients without a usual source of PHC or whose point of entry into the investigation process was not their usual source of PHC also experienced shorter investigation-to-diagnosis times suggests, as well, that using a faster channel (e.g., emergency room, walk-in clinic) can provide more rapid access to diagnostic technologies or specialized services. More specifically, patients with lung cancer reported shorter investigation-to-diagnosis times than did patients with breast cancer. Even though a lung X-ray (which is, in fact, easily accessible) alone does not confirm lung cancer, it may be that the moment when the physician discussed this test result with them was often interpreted by patients as the moment when the cancer diagnosis was pronounced. For breast cancer, patients may have been less quick to consider the results of the first examination as the moment when the diagnosis was made, given the high frequency of false positives, such that supplementary testing is required. It may also be that symptom urgency and the unfavorable prognosis of lung cancer prompted faster diagnostic procedures in those cases than in breast cancer, since as mentioned by Tørring et al. [13] more rapid investigations are generally undertaken for the sickest patients, leading to very short times to diagnosis.

### 4.3. Presence of Metastatic Cancer at Time of Diagnosis

As expected given the inherent nature of those diseases, our results indicated that patients with lung or colorectal cancer were more likely to have metastases at the time of diagnosis. Aside from the cancer site, another factor that might conceivably increase the risk of finding metastases at the time of diagnosis would be length of time between onset of illness and its diagnosis. However, our results did not show any significant association between the length of the prediagnostic period and the presence of metastases at time of diagnosis; this may be due, at least in part, to a limitation in the statistical power of our analyses. Besides, as pointed out by Neal [2], cancer progression also depends largely on the aggressiveness of the tumour, as a more aggressive tumour can produce more rapidly changing symptoms and prompt faster diagnosis (and thus a shorter time to diagnosis), while leading to a more negative outcome (advanced stage at diagnosis). Conversely, a tumour that progresses slowly can cause less specific symptoms and take longer to diagnose, without necessarily reaching an advanced stage by the time of diagnosis. Tørring et al. [13] showed, in fact, that very short times to diagnosis could be associated with higher mortality levels, as more rapid investigations are generally undertaken for the sickest patients. Such cases are likely to influence the strength of the observed association between elapsed time and the presence of advanced cancer at time of diagnosis.

Lastly, continuity of care at the usual source of PHC did not appear to be closely linked with the choice of point of entry into the cancer investigation process or with elapsed time. Hansen et al. [31] noted, in fact, that elapsed time between first symptoms and primary care consultation was not associated with better knowledge of the patient by the physician and that investigation-to-diagnosis times were shorter among patients seen by physicians who knew them less well. These results suggest that, in care-seeking for the investigation of potentially serious symptoms, care continuity, supported by the quality of the patient-physician relationship, is not enough in itself and must be paired with timely accessibility of care and a wide range of available services. In this regard, organizational attributes that foster accessibility and comprehensiveness (e.g., longer opening hours, technical platform, multidisciplinary team, information technologies, and walk-in consultations), which have been put forward in the primary care medical services models advocated by recent reforms in Quebec [43] and in other Canadian provinces [44], are likely to be a promising development in the use of services for health conditions such as cancer. While the impact of new PHC organizational models in Quebec (family medicine groups, network clinics) on patients' experience of care has not yet been clearly demonstrated [45], our studies indicate that these models have organizational attributes (e.g., increased hours of service, a mix of scheduled and walk-in consultations, wider range of services, and easier access to technical platforms and secondary care services) that foster accessibility and comprehensiveness of care [45–47], which appear to be important in the cancer care-seeking process.

### 4.4. Limits

While the associations may actually be very weak between experience of care at the usual source of PHC and time to cancer diagnosis and between time to diagnosis and presence of metastases at time of initial diagnosis, the fact that the associations shown in our results are rather modest and do not always meet the threshold of statistical significance probably reflects lack of power in our analyses. Moreover, since this exploratory study examined a phenomenon that presents considerable variability, our sample size proved to be relatively small. Our inclusion in the sample of patients with cancers that differed in terms of clinical onset, evolution, and investigation process also probably contributed to greater variability in the data. Given the size of the subsamples for each cancer site, stratified analyses by cancer site were not feasible. Moreover, given the cross-sectional nature of our design, caution is advised in attempting to draw causal inferences from our data. In this regard, the possibility of a reverse causation in the associations between comprehensiveness of care and shorter symptoms-to-investigation time cannot be excluded. As comprehensiveness was measured after diagnosis, it is possible that patients who were investigated promptly rated their PHC provider more highly in terms of comprehensiveness of care because they had a good experience with their cancer diagnosis process at their usual source of PHC.

It is not possible, from our data, to ensure that our respondents are representative. It could be that recruiting in oncology clinics favored the participation of patients whose health status was relatively good and that more seriously ill patients were underrepresented in our sample. Also, our study was based on survey data collected from patients, which may be subject to certain biases. Respondents' perceptions may have been influenced by memory bias, in particular with regard to the dates sought in the questionnaire. This bias probably contributed to the lack of precision in measurements of time to cancer diagnosis, especially given that our questions concerned sensitive events (e.g., date when cancer symptoms first appeared, date when medical services were obtained, and date when the diagnosis was disclosed). It is also possible that some dates (e.g., date when the physician prescribed the tests to investigate the cancer, date when the patient was told about the cancer diagnosis) were interpreted differently from one patient to another. Neal et al. [10] underscored the difficulty of obtaining specific dates from patients, as well as the widely variable results obtained in studies on elapsed time in cancer diagnosis [2]. As in other studies [10, 15], the date of first symptoms appearance was the item with the highest proportion of imprecise data in our study. Developing data collection tools that could measure this elapsed time with greater validity [14] would improve the quality of research in this area. However, beyond memory bias and regardless of the methodology adopted, it will probably always be difficult to capture the complex and intimate nature of individuals' recognition of the symptoms that prompted them to consult a healthcare professional [9]. This difficulty extends well beyond the simple detection by patients of more or less specific signs and symptoms, not to mention the difficulties at the purely medical level of making the connection between these symptoms and cancer [2, 9].

Despite these limitations, in a patient-centred approach, we preferred to use information collected from patients as our source of data for this exploratory study, as we felt it was more likely to provide a better idea of elapsed times as perceived by patients than would have been obtained from physician surveys or from data in patients' records [2]. It should be noted that times measured using patient questionnaires are generally shorter than those measured using in-depth qualitative interviews [2]. However, the objective of our study was not to describe elapsed times in cancer diagnosis but rather to verify the association between the experience of care in primary care and the choice of point of entry into the investigation process, the elapsed time between key events, and, finally, the presence of advanced-stage cancer at time of diagnosis. In our study, the impact of the lack of precision in measuring elapsed times was probably an underestimation of the observed associations.

Other measurements may also have introduced a certain lack of precision into our data. For instance, symptom urgency was determined, by the physicians consulted, from patients' responses to an open-ended question about the nature of their symptoms. The quality of this data thus depended largely on details provided by patients in the questionnaire but also reflected the physicians' views of the urgency of the patients' condition, which could differ from those of the patients. Lastly, although our experience of care measurement was based on validated questions, which were included in a large-scale survey recently carried out in Quebec by the Institut de la Statistique du Québec [48], there is no universally accepted measure for experience of care, and thus our results apply specifically to the way in which we constructed our indices of accessibility, continuity, and comprehensiveness.

## 5. Conclusion

The type of cancer and the nature of the symptoms and their evolution are key determinants of the choice of point of entry into the cancer investigation process, time to diagnosis, and presence of metastasis at time of diagnosis. The results of our study suggest, however, that patients' experience of care at their usual source of primary care, particularly with regard to accessibility and comprehensiveness, exerts a certain influence on their choice of point of entry into the investigation and on time to diagnosis. Within the context of current health services reforms, focusing on models of primary care services organization that foster accessibility and comprehensiveness of care would be a worthwhile avenue to consider in relation to patients' use of cancer diagnostic services.

## Figures and Tables

**Table 1:** Patient characteristics by cancer site.

	All patients		Patients with breast cancer		Patients with lung cancer		Patients with colorectal cancer		Difference between cancer sites
	(*n* = 307)		(*n* = 107)		(*n* = 91)		(*n* = 109)		
	*n*	%	*n*	%	*n*	%	*n*	%	*p* value^*∗*^
Female	213	69.4	106	99.1	59	64.8	48	44.0	0.003
Age = 65 years or more	111	36.2	23	21.5	40	44.0	48	44.0	<0.001
College or university education	174	56.7	68	63.6	41	45.1	65	59.6	0.024
Income adjusted for household size (top quartile)	59	23.0	23	24.0	12	17.1	24	26.4	0.369
At least one cardiometabolic risk factor^1^	148	48.4	42	39.3	46	51.1	60	55.1	0.056
At least one chronic illness^2^	153	50.0	53	49.5	58	64.4	42	38.5	0.001
Presence of metastasis at initial diagnosis	84	27.9	6	5.7	42	47.7	36	33.3	<0.001

^1^Cardiometabolic risk factors: high blood pressure, diabetes, and hypercholesterolemia.

^2^Chronic illnesses: cardiac, respiratory, musculoskeletal, digestive, mental health, or stroke-related problems.

^*∗*^Pearson's Chi-Square test. For gender, we compared patients with lung cancer to patients with colorectal cancer (patients with breast cancer were excluded).

**Table 2:** Characteristics of primary care management.

	All patients	Patients with breast cancer	Patients with lung cancer	Patients with colorectal cancer	Difference between cancer sites
	(*n* = 307)	(*n* = 107)	(*n* = 91)	(*n* = 109)	*p* value^*∗*^
Having a usual source of PHC (%)	89.3	86.9	93.4	88.1	0.301
Accessibility^1^ (mean score on 10)	6.8	6.5	7.1	6.9	0.279
Continuity^1^ (mean score on 10)	8.0	7.6	8.6	7.8	0.053
Comprehensiveness^1^ (mean score on 10)	7.8	7.7	8.3	7.4	0.115

^1^Scores of experience of care relate to care received at the usual source of PHC in the two years preceding the cancer diagnosis. A score of 0 was attributed to patients who had no usual source of PHC.

^*∗*^Pearson's Chi-Square test for comparison of %, and Fisher's test for comparison of mean scores.

**Table 3:** Point of entry into the cancer investigation process.

	All patients	Patients with breast cancer	Patients with lung cancer	Patients with colorectal cancer	Difference between cancer sites
	(*n* = 307)	(*n* = 107)	(*n* = 91)	(*n* = 109)	
	%	%	%	%^*∗*^	*p* value^*∗*^
Usual source of PHC	46.9	43.9	51.7	45.9	0.535
Another PHC clinic	10.1	14.0	9.9	6.4	0.179
Emergency room	17.9	3.7	24.2	26.6	<0.001
Specialist	16.3	20.6	13.2	14.7	0.320
Other or no response	8.8	17.8	1.1	6.4	<0.001
Total	100	100	100	100	

^*∗*^Pearson's Chi-Square test.

**Table 4:** Elapsed times (median; in days) in cancer diagnosis.

	All patients	Patients with breast cancer	Patients with lung cancer	Patients with colorectal cancer	Difference between cancer sites
	(*n* = 307)	(*n* = 107)	(*n* = 91)	(*n* = 109)	*p* value^*∗*^
Symptoms-to-investigation time	47	41	40	64	0.143
Time between first symptoms and making appointment^1^	21	18	16	30	0.749
Time between making appointment and start of investigation^1^	11	10	9	12	0.593
Investigation-to-diagnosis time	32	46	29	24	0.092

^1^Times between first symptoms and making appointment for these symptoms and between making appointment for the first symptoms and start of investigation could not be calculated in 28% of cases, because of the large amount of missing data for the date when the first appointment was made after noticing symptoms (24%).

^*∗*^Median test.

**Table 5:** Factors associated with patients' use of their usual source of PHC as point of entry into the cancer investigation process. Patients with a usual source of PHC (*n* = 274).

	OR	CI 90%	*p* value
Cancer site (ref.: breast)			
Lung	1.07	[0.59–1.94]	0.844
Colorectal	0.77	[0.41–1.45]	0.500
Symptom urgency (ref.: yes)			
No	7.02	[3.93–12.53]	<0.001
Sex (ref.: male)			
Female	0.74	[0.42–1.29]	0.370
Age^1^	0.98	[0.96–1.00]	0.157
Education level (ref.: primary or secondary)			
College or university	1.08	[0.68–1.72]	0.779
Accessibility at the usual source of PHC^1^	1.03	[0.90–1.17]	0.722
Continuity at the usual source of PHC^1^	0.92	[0.74–1.15]	0.539
Comprehensiveness at the usual source of PHC^1^	1.25	[1.06–1.46]	0.023

^1^In this model, age and experience of care indices are continuous variables.

Note: results were similar for models including level of morbidity or type of PHC clinic as covariates and for models excluding symptom urgency.

**Table 6:** Factors associated with symptoms-to-investigation and investigation-to-diagnosis times (*n* = 244).

	First symptoms-to-investigation time		Investigation-to-diagnosis time	
	Hazard ratio^2^	*p* value	Hazard ratio^2^	*p* value
Cancer site (ref.: breast)				
Lung	1.03	0.868	1.46	0.035
Colorectal	0.94	0.745	1.28	0.179
Symptom urgency (ref.: yes)				
No	0.82	0.220	0.92	0.598
Sex (ref.: male)				
Female	1.14	0.440	1.06	0.724
Age^1^	1.01	0.268	1.00	0.762
Education level (ref.: primary or secondary)				
College or university	1.05	0.744	1.03	0.808
Accessibility at the usual source of PHC^1^	1.06	0.223	1.13	0.004
Continuity at the usual source of PHC^1^	0.95	0.485	1.00	0.997
Comprehensiveness at the usual source of PHC^1^	1.11	0.052	1.01	0.828
Correspondence between point of entry into investigation and usual source of PHC (ref.: usual source of PHC = point of entry)				
Usual source of PHC ≠ point of entry	0.91	0.542	1.40	0.023
No source of PHC	1.71	0.473	4.00	0.036

^1^In these models, age and experience of care indices are continuous variables.

^2^For the Cox regression regarding first symptoms-to-investigation time, the origin of the time-scale of the analysis was the date of first symptoms and the exit point was the start of investigation. For the analysis of investigation-to-diagnosis time, the origin of the time-scale of the analysis was the start of the investigation and the exit point was the disclosure of the diagnosis. A hazard ratio greater than one indicates a shorter time, that is, that the event (investigation, for the symptoms-to-investigation time, and diagnosis, for the investigation-to-diagnosis time) occurred more rapidly.

Note: results were similar for models including level of morbidity or type of PHC clinic as covariates, for models excluding symptom urgency, and for analyses performed for the subgroup of patients who had used their usual source of PHC as point of entry into cancer investigation.

**Table 7:** Factors associated with having metastases at initial cancer diagnosis (*n* = 238).

	OR	CI 90%	*p* value
Age	1.01	[0.98–1.03]	0.757
Cancer site (ref.: breast)			
Lung	16.96	[6.45–44.62]	<0.001
Colorectal	7.48	[2.85–19.66]	0.001
Symptoms-to-investigation time^1^	1.05	[0.99–1.11]	0.155
Investigation-to-diagnosis time^1^	0.90	[0.79–1.03]	0.195

^1^In this model, elapsed times are continuous variables.

**Table 8:** Proportion (%) of attributed and missing data in dates used to calculate elapsed times (Appendix A).

	All patients		Breast cancer		Lung cancer		Colorectal cancer	
	(*n* = 307)		(*n* = 107)		(*n* = 91)		(*n* = 109)	
	Attributed data^*∗*^	Missing data	Attributed data^*∗*^	Missing data	Attributed data^*∗*^	Missing data	Attributed data^*∗*^	Missing data
Date of first symptoms	52	13	52	17	43	12	58	11
Date of scheduling appointment	39	24	38	23	34	26	45	22
Date of start of investigation	37	11	28	12	32	9	50	13
Date of diagnosis	34^1^	0	20^2^	0	33^3^	0	48^4^	0

^*∗*^Patients who were unable to provide specific dates were asked to indicate the month and year of the event and to specify whether the event occurred at the beginning, middle, or end of the month; the date corresponding to the mid-point of that period was then attributed to the event. Residual missing data for date of diagnosis were estimated from the dates of diagnosis entered in the oncology clinics' cancer registries. However, since self-reported dates of diagnosis usually differed from registries' dates of diagnosis, we replaced missing data in questionnaire by the data of the registries, adjusted by the mean difference between the dates of diagnosis entered in the registries and the dates indicated by responding patients, by cancer site.

^1^This proportion includes 26% from partial data provided by patients and 8% from the cancer registries.

^2^This proportion includes 17% from partial data provided by patients and 3% from the cancer registries.

^3^This proportion includes 23% from partial data provided by patients and 10% from the cancer registries.

^4^This proportion includes 36% from partial data provided by patients and 12% from the cancer registries.

**Table 9:** Composition of indices of experience of care at the usual source of primary care (Appendix B).

Experience of care dimension	Questions/statements	Scale
Accessibility	In this place, if your doctor (the one who looks after your care) is not available, you can see another doctor.	Always, often, sometimes, never
	When you need to see a doctor at this place, in general, how long do you have to wait to see that doctor by appointment?	Less than 2 weeks, 2 to 4 weeks, 1 to 3 months, 4 months or more
	When you need care quickly or urgently, how long does it take for you to be able to see a doctor at this place?	Less than 24 hours, 1 to 2 days, 3 to 4 days, 5 days or more

Continuity	How long have you been going to this place?	Less than 2 years, 2 to 5 years, 6 to 9 years, 10 years or more
	When you go to this place, you see the same physician.	Always, often, sometimes, never
	*In this place,…*	
	they know your medical history	Very much, moderately, somewhat, not at all
	they know all the prescription medicines you are taking	
	you can be followed for a chronic condition	

Comprehensiveness	*In this place,…*	
	they look after all your health problems, whether physical or psychological	Very much, moderately, somewhat, not at all
	when you go for a visit, the doctor takes the time to talk about prevention and asks about your lifestyle habits	
	they help you to get all the health services you need	
	they take into account your opinion and your wishes regarding the services they offer	
	they help you to evaluate the pros and cons when you need to take a decision concerning your health	

## Appendices

### A. Proportion of Attributed and Missing Data in Dates Used to Calculate Elapsed Times

See Table 8.

### B. Composition of Indices of Experience of Care at the Usual Source of PHC

See Table 9.
